# Self-Healing of Microcracks in Engineered Cementitious Composites (ECC) Under a Natural Environment

**DOI:** 10.3390/ma6072831

**Published:** 2013-07-15

**Authors:** Emily N. Herbert, Victor C. Li

**Affiliations:** Department of Civil and Environmental Engineering, University of Michigan, Ann Arbor, MI 48109, USA; E-Mail: eherbert@umich.edu

**Keywords:** engineered cementitious composites (ECC), concrete, self-healing

## Abstract

This paper builds on previous self-healing engineered cementitious composites (ECC) research by allowing ECC to heal outdoors, in the natural environment, under random and sometimes extreme environmental conditions. Development of an ECC material that can heal itself in the natural environment could lower infrastructure maintenance costs and allow for more sustainable development in the future by increasing service life and decreasing the amount of resources and energy needed for repairs. Determining to what extent current ECC materials self-heal in the natural environment is the first step in the development of an ECC that can completely heal itself when exposed to everyday environmental conditions. This study monitored outdoor ECC specimens for one year using resonant frequency (RF) and mechanical reloading to determine the rate and extent of self-healing in the natural environment. It was found that the level of RF, stiffness, and first cracking strength recovery increased as the duration of natural environment exposure increased. For specimens that underwent multiple damage cycles, it was found that the level of recovery was highly dependent on the average temperature and amount of precipitation between each damage event. However, RF, stiffness, and first cracking strength recovery data for specimens that underwent multiple loading cycles suggest that self-healing functionality can be maintained under multiple damage events.

## 1. Introduction

Cracks are unavoidable during the lifetime of a concrete structure. Structures directly exposed to the natural environment are susceptible to cracking not only from factors such as excessive loading and restrained shrinkage, but also from harsh environmental conditions. Cracks negatively impact concrete structures in numerous ways. Cracking may weaken a structure by negatively impacting the mechanical properties, or it may lower the durability by creating pathways for harmful agents to penetrate the structure and attack the reinforcing steel or surrounding concrete. This increases the maintenance costs of concrete structures and decreases the service life. Therefore, the development of a concrete that can regain this loss of performance due to cracking is highly desirable.

Studies have shown that cracked concrete has the ability to heal itself over time when exposed to water. It has been found that there is a gradual reduction in permeability of damaged concrete as water is allowed to flow through the cracks. This decrease in permeability is due to diminishing crack widths as healing occurs [1,2,3]. Healing has been shown to be a complex process involving several chemical and physical mechanisms. The following mechanisms for healing have been cited by previous research: hydration of unreacted cement, swelling of C–S–H, precipitation of calcium carbonate crystals, closing of cracks by impurities within the water, and the closing of cracks by concrete particles from spalling on the crack faces. Although all of these mechanisms contribute to healing, it has been shown that the precipitation of calcium carbonate is the main mechanism for the self-healing of cracks in concrete [1].

The extent of self-healing in cracked concrete was found to be highly dependent on the crack width, with smaller cracks healing more completely and at a faster rate than larger cracks. In some cases with small crack widths, cracks may heal completely, thus increasing the durability and potentially the mechanical properties of the damaged material [1,4]. However, this is rare since most concrete materials are brittle and incapable of achieving crack widths small enough to undergo self-healing.

A number of approaches to induce self-healing in concrete have been attempted. Of these approaches, the most common are: chemical encapsulation [5], bacterial encapsulation [6,7,8], mineral admixtures [9,10,11,12,13,14], chemicals in glass tubing [15,16,17], and intrinsic healing with self-controlled tight crack widths [18,19,20,21]. A review of the advances and limitations of these approaches can be found in Li and Herbert [22]. Chemical encapsulation utilizes self-healing chemical agents contained in microcapsules, which are dispersed uniformly within concrete and fracture to release a healing agent when a crack occurs. The bacterial encapsulation technique is similar, but bacteria that induce precipitation of calcium carbonate are used as the self-healing agent. Some mineral admixture approaches utilize expansive agents and geo-materials dispersed within a concrete matrix, which expand to fill cracks when damage occurs, while other approaches utilize the addition of fly ash and blast furnace slag. Glass tubing is considered an alternative approach to chemical encapsulation since a self-healing chemical agent is used, but it is stored within the concrete matrix in glass tubes instead of microcapsules. Intrinsic healing with self-controlled tight crack widths utilizes continued hydration of unreacted cement, pozzolanic reactions, and carbonation to produce C–S–H and calcium carbonate within cracks that are less than 100 µm in width. Among these promising techniques, the intrinsic healing with self-controlled tight crack width approach is one that has clearly demonstrated the ability to achieve both water permeability reduction and recovery of mechanical properties [18,19,20,21].

A variety of observation methods have been used for detecting self-healing in concrete materials. Amongst these are scanning electron microscopy (SEM) [5,7,8,9,18,23], energy dispersive spectroscopy (EDS) [5,7,9,19,23], Fourier transform infrared spectroscopy (FTIR) [7,23], permeability tests [10,11,12,13,17,19], and X-ray computed tomography [17,24]. In most cases, the observation methods reveal self-healing products that seal the crack, but that may or may not imply recovery of mechanical properties. In this study, a combination of resonant frequency measurement changes and direct tension reloading [19,20,21] are adopted to detect self-healing. The recovery of cracking strength and material stiffness are proof of self-healing in a mechanical sense, beyond self-sealing.

**Figure 1:**
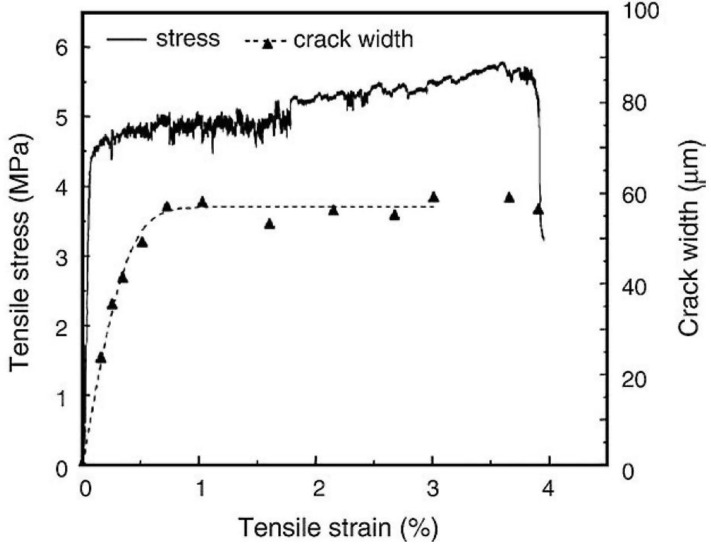
Typical engineered cementitious composites (ECC) stress-strain-crack width curve [25].

ECC is a fiber reinforced cementitious composite that has been optimized through the use of micromechanics to achieve high tensile ductility and tight crack widths. As seen in Figure 1, ECC has the ability to reach tensile strain capacities of 3%–5% under loading (compared with 0.01%–0.02% for normal concrete) while maintaining tight cracks widths of less than 60 μm [25,26,27].These tight crack widths are an intrinsic material property of ECC and promote robust self-healing behavior that is not easily attainable in brittle concrete with uncontrolled crack widths. The tight crack width of ECC does not depend on any amount of steel reinforcement, and also does not depend on the size-scale of the specimen or structure.

Self-healing has been shown to be extensive and reliable in ECC when specimens are healed under controlled laboratory conditions. ECC specimens have regained both permeability and mechanical properties under a variety of healing regimens and harsh conditions, including chloride and highly alkaline environments [18,19,20,21]. The healing products have also begun to be characterized and it has been found that both precipitation of calcium carbonate and the hydration of unreacted cement play an important role in the healing process in ECC [23].

While extensive research has been done on the self-healing of ECC under controlled laboratory conditions, only one study has been completed on the self-healing of ECC in the natural environment. That study was a short-term investigation that showed that self-healing of ECC in the natural environment is feasible [28]. This paper expands on the previous natural environment research by monitoring outdoor ECC samples for one year using resonant frequency and mechanical reloading. The objective of this research is to establish the foundation for exploitation of the self-healing functionality of ECC infrastructure in field applications.

## 2. Experimental Investigation

### 2.1. Mix Proportion and Raw Materials

The self-healing properties of an ECC mix are highly dependent on the crack width of the material. For this reason, it was decided to use an ECC mix with smaller crack widths than the mix used in the previous natural environment study [28]. The mix proportions used in this study are given in Table 1. This mix had average crack widths of 24 and 30 µm at 0.5% and 1.0% tensile strain, while the mix used in the previous study had crack widths of 36 and 44 µm at the corresponding imposed strains [29]. The crack widths reported here are those of specimens after unloading. During load, the crack widths are typically larger by about 15% [19].

**Table 1:** Mix proportions of engineered cementitious composites (ECC).

Component	Cement	Fly Ash	Sand	Water	HRWRA ^a^	Fiber
Weight Fraction	1	2.2	1.16	0.85	0.01	0.067

^a^ HRWRA: High Range Water Reducing Admixture

The raw materials used in the ECC mix were: Type I ordinary portland cement, Class F fly ash, silica sand with an average particle size of 110 µm, a polycarboxylate-based high range water reducing admixture (HRWRA), and polyvinyl alcohol (PVA) fibers. The PVA fibers accounted for 2% of the total mix volume and were 12 mm in length with an average diameter of 39 µm. The fibers had a tensile strength of 1600 MPa, a density of 1300 kg/m^3^, an elastic modulus of 42.8 GPa, and a maximum elongation of 6%. Also, the surfaces of the fibers were coated with an oiling agent (1.2% by weight) to reduce the interfacial chemical bond between the fiber and matrix caused by the strong hydrophilic nature of the PVA fibers [25,30].

### 2.2. Specimen Preparation and Preloading

After mixing, the fresh ECC mix was cast into molds measuring 300 mm × 76 mm × 12.5 mm and covered in plastic sheeting. After 24 h, the specimens were removed from the molds and cut to 200 mm in length to minimize edge effects and bending stresses caused during tensile loading. Specimens were then air cured at room temperature in the laboratory until testing. The day prior to testing, aluminum plates were glued to both ends of the specimens to facilitate gripping within the load frame.

A total of 60 specimens were prepared, and these were then separated into four sets of 15 specimens each. Each set contained 5 control specimens, 5 specimens that were preloaded to 0.5% tensile strain, and 5 specimens that were preloaded to 1.0%. These strain values were chosen based on the previous natural environment study [28] and to produce samples to simulate varying degrees of tension-induced damage in an actual structure. Note that these imposed deformations are high when compared with the failure strain of normal concrete at 0.01%–0.02%. All preloading was carried out 7 days after the specimens were casted using uniaxial tensile loading. Loading was applied using a load frame with a 25 kN capacity under displacement control and a loading rate of 0.5 mm/min. Two Linear Variable Displacement Transducers (LVDTs) were mounted to the specimens during loading to measure the tensile elongation. When the tensile strain reached the desired value, the load was released and the specimens were removed from the loading frame.

### 2.3. Natural Environment Exposure

After preloading, specimens were placed outdoors in a location where they would be fully exposed to natural environmental conditions. This study took place in Ann Arbor in Southeast Michigan over a 12-month period from September 2011 through September 2012, so the specimens were exposed to a wide range of temperatures (−11.7 to +32.8 °C) and precipitation events.

### 2.4. Self-Healing Evaluation Methods

#### 2.4.1. Resonant Frequency (RF)

RF measurements based on ASTM C215 for the longitudinal mode were used to monitor the rate of self-healing in the preloaded specimens. Although the RF measurement technique is commonly used to evaluate concrete damage after exposure to freezing and thawing cycles, it has proven to be a useful method for determining the rate and extent of self-healing in ECC [19]. RF values were determined before and after preloading to quantify the amount of damage, and then once a week throughout the duration of the study to evaluate the rate of self-healing. The RF value of a specimen was calculated as the average of 3 consecutive RF measurements. 

#### 2.4.2. Uniaxial Tensile Test (Reloading)

**Figure 2:**
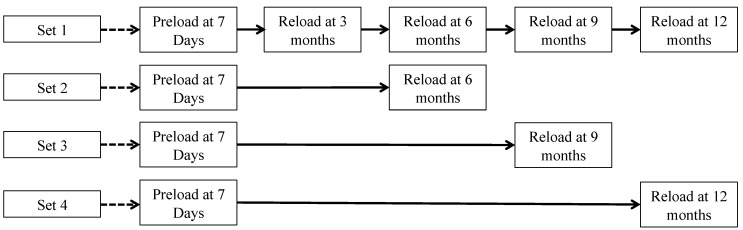
Preloading and reloading schedule. Length of solid arrows schematically illustrates the exposure time to the natural environment prior to the next reloading event.

To assess the robustness of the self-healing products, mechanical properties of the specimens were measured once they were allowed to heal in the natural environment. After a given exposure time period for healing, specimens were reloaded using uniaxial tensile tests and the mechanical properties were compared to those measured during preloading to determine the level of recovery. The exposure times of 3, 6, 9 and 12 months for Specimen Set 1, Set 2, Set 3 and Set 4, respectively, are schematically illustrated in Figure 2. For Specimen Set 1, three additional reloading events were applied to examine the influence of repeated damage on the ability of the material to maintain self-healing functionality. All reloading events were conducted using the same method described above for preloading, where specimens preloaded to 0.5% and 1.0% tensile strain were reloaded to the same strain magnitudes. Thus the reloading events in this study were used both as a method of assessing the rehealing of mechanical properties of ECC (for all four Sets), as well as to serve as additional damage causing events (for Set 1).

## 3. Experimental Results and Discussion 

### 3.1. Resonant Frequency Recovery

**Figure 3:**
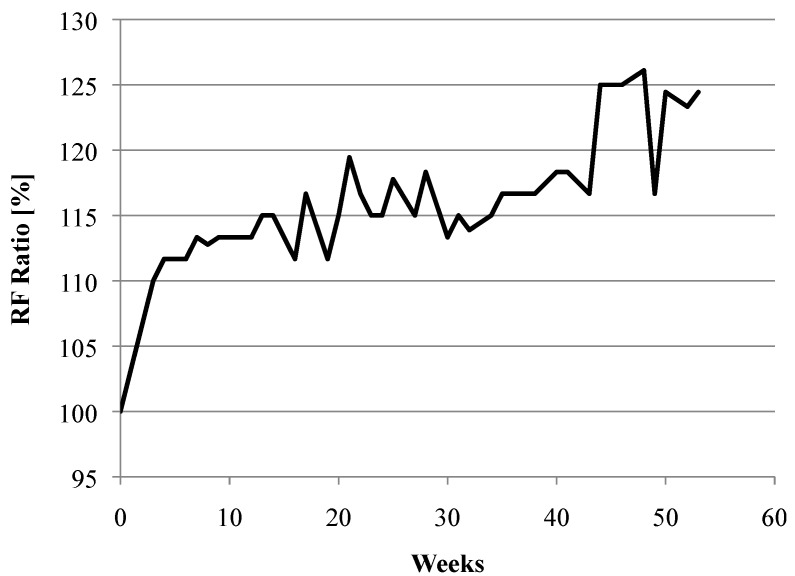
Example of RF for specimens with no cracks (not preloaded) exposed to a natural environment. The general increasing RF trend is due to continued hydration of the bulk material.

In addition to self-healing of microcracks, continued hydration over time in the bulk material also increases the RF values. Figure 3 shows this increase by plotting the RF ratio of a control sample (with no preloading), which was exposed to the same environmental conditions as the preloaded specimens. The RF Ratio shows the change in RF of the control sample and is calculated by

(1) $$\text{RF Ratio = }\frac{RF_{environment}}{RF_{original}}$$

where *RF_original_* is the RF value of the specimen before it is exposed to the natural environment and *RF_environment_* is the RF value of the specimen during its natural environment exposure.

To highlight the effect of microcrack healing, and to account for the effects of continued hydration which also increases the measured resonant frequency, RF values for preloaded specimens were normalized, so that

(2) $$\text{Normalized RF = }\frac{RF_{preload,environment}}{RF_{control,environment}}$$

where *RF_preload,environment_* is the RF value of preloaded specimens that were exposed to the natural environment and *RF_control,environment_* is the RF value of control specimens that underwent the same environmental exposure. The Normalized RF removes the effect of bulk material hydration over time and provides RF values that are due solely to self-healing of microcracks. Therefore, a Normalized RF value of 100% implies that a sample has fully healed from any damage that was induced during preloading. 

#### 3.1.1. Effect of Natural Environment Exposure Time on RF

**Figure 4:**
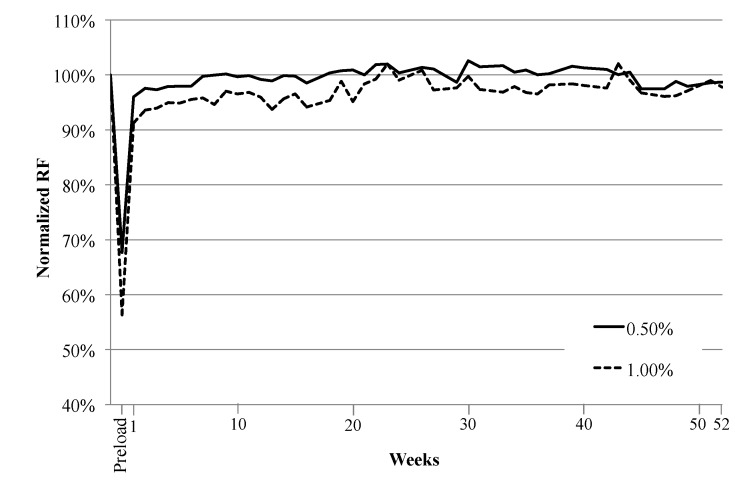
Effect of natural environment exposure time on recovery of RF. Data is for specimens exposed to the natural environment for 12 months, and each data line is an average of 5 specimens.

The RF recovery of specimens preloaded to 0.5% and 1.0% was quite rapid once they were exposed to the natural environment, as shown in Figure 4 (Figure 4 is based on data from Specimen Set 4 with the longest exposure time. Data from Specimen Sets 1–3 (not shown for clarity) essentially trace the same curve shown in Figure 4 up to the time when these specimens underwent reloading). Due to the formation of cracks within a specimen during loading, there was a large drop in RF values after the specimens were preloaded, yet the Normalized RF recovered between 90% and 96% after the first week of natural environment exposure. This is similar to results seen under laboratory conditions where the largest RF recovery occurred after a specimen’s first exposure to water [19,23,31]. However, during the previous natural environment study it was found that the RF recovery was more gradual than that seen under laboratory conditions [28]. Therefore, it can be assumed that the rate of RF recovery in the natural environment varies depending on weather conditions (precipitation, temperature, *etc.*).

The rate of RF recovery drastically decreased after the first week of natural environment exposure, but the Normalized RF values remained relatively stable between 95% and 105% (Figure 4). This is similar to results seen in past laboratory [19,23] and natural environment studies [28]. The stabilized Normalized RF values do not appear to be altered by the time length of exposure to the natural environment.

The fluctuations in RF values between 95% and 105% shown in Figure 4 could be due to the washout and reformation of healing products after precipitation events. Similar fluctuations were seen in the previous natural environment study, along with visual observations showing the changes in healing products [28]. Calcium hydroxide (Ca(OH)_2_), formed during the hydration reaction when cracks are exposed to precipitation, is easily dissolved in water and could be dissolving during rainfall events. Therefore, a sudden drop in RF could be due to the dissolution of Ca(OH)_2_ during one rainfall event, and a sudden increase could be due to the reformation of Ca(OH)_2_ after the next rainfall. Typically the large fraction of fly ash in the ECC mix would consume Ca(OH)_2_ during the pozzolanic reaction, but the kinetics of this reaction are slow, and may not be occurring fast enough during each precipitation event to transform the Ca(OH)_2_ into the more stable C–S–H gel.

#### 3.1.2. Effect of Multiple Damage (Reloading) Events on RF

**Figure 5:**
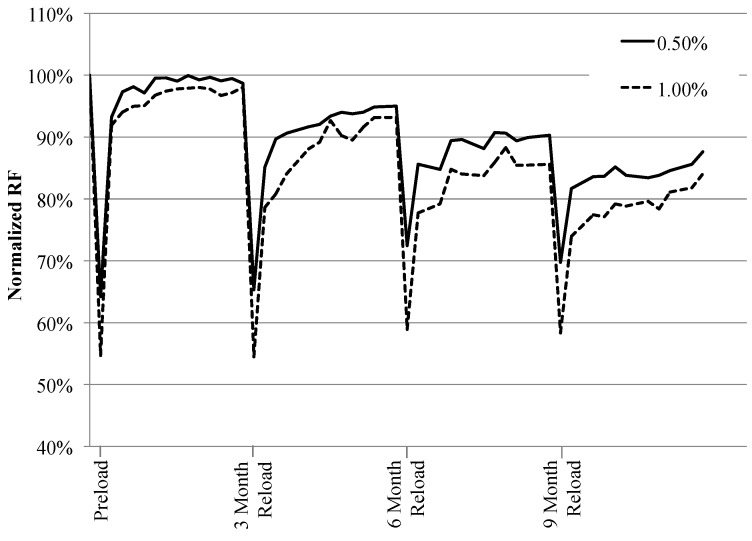
Effect of multiple damage (reloading) events on recovery of RF. Each data line is an average of 5 specimens.

While the amount of RF recovery decreased after each loading cycle (in Specimen Set 1), the specimens recovered to reasonable RF values even after three reloading events (Figure 5). There was a drastic drop in RF values after each reloading event due to the reopening of healed cracks and the formation of new cracks within the specimens, yet the values recovered significantly even after only one additional week of natural environment exposure. After the third loading cycle, the 0.5% specimens were able to recover to a Normalized RF value of 88% and the 1.0% specimens recovered to 84%. This shows that self-healing in ECC is relatively repeatable, despite deterioration in the magnitude of recovery with each damage cycle.

### 3.2. Stiffness and First Cracking Strength Recovery

To determine the level of stiffness recovery in a self-healed specimen, the stiffness of the specimen measured during reloading was compared to the stiffness value measured during preloading. The secant stiffness was measured between 0.5 and 2.0 MPa and the stiffness recovery was calculated using

(3) $$\text{Stiffness Recovery = }\frac{Stiffness_{reload}}{Stiffness_{preload}}$$

where *Stiffness_reload_* is the secant stiffness of the specimen during reloading and *Stiffness_preload_* is the secant stiffness of the specimen during preloading. 

The recovery of first cracking strength was also determined by comparing the value measured during reloading with the value measured during preloading. Following the JSCE design recommendation [32], the first cracking strength was defined as the stress value when the first crack was initiated under tensile loading, where the assumption of linear elasticity could no longer hold. The first cracking strength recovery was then calculated using

(4) $$\text{First Cracking Strength Recovery = }\frac{Strength_{reload}}{Strength_{preload}}$$

where *Strength_reload_* is the first cracking strength of the specimen during reloading and *Strength_preload_* the first cracking strength of the specimen during preloading.

#### 3.2.1. Effect of Natural Environment Exposure Time on Mechanical Properties

**Figure 6:**
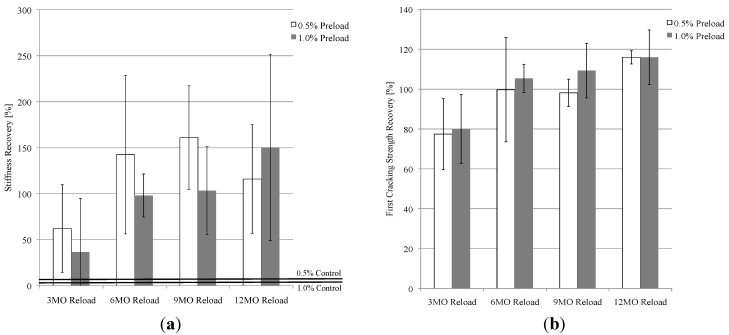
Effect of natural environment exposure time on recovery of mechanical properties. Recovery of (**a**) stiffness and (**b**) first cracking strength. Each data bar is an average of 5 specimens.

On reloading, it was found that the level of stiffness recovery increased as the duration of natural environment exposure (3, 6, 9, and 12 months) of the preloaded specimens increased (Figure 6a). The specimens reloaded at 3 months had regained, on average, 62% of their initial stiffness value when preloaded to 0.5%. This is similar to results seen in the previous natural environment study, where specimens preloaded to 0.5% were regaining up to 65% of their initial stiffness after 3 months of exposure [28]. The level of stiffness recovery continued increasing with the amount of environmental exposure, with stiffness recoveries often greater than 100% after 6 months. This level of recovery has also been seen in previous work where specimens have been healed under laboratory conditions [20]. The stiffness “recovery” of control specimens preloaded to 0.5% and 1.0% and immediately reloaded without allowing any self-healing to occur was also calculated and is shown in Figure 6a. These serve as references for the actual stiffness recovery data observed in specimens exposed to the natural environment.

Stiffness recovery can be attributed to both the healing of microcracks and the increase in stiffness of the bulk matrix material due to continued hydration. As seen by the control specimens in Figure 6a, the stiffness of specimens upon reloading is quite low when no self-healing occurs. This is due to the fact that microcracks reopen upon reloading with little resistance by the partially debonded bridging fibers that are not tightly stretched due to incomplete retrieval of the fibers into the matrix tunnel. If there were no healing of the microcracks in specimens exposed to the natural environment, the composite stiffness would remain low even if there were continued hydration of the bulk material since the low stiffness of the crack planes would dominate the overall stiffness (analogous to a number of low stiffness (unhealed cracks) and high stiffness (bulk material) springs connected in series). Thus the observed significant increase in composite stiffness of the specimens exposed to the natural environment over those of the control specimens confirms that the microcracks did heal. However, since recoveries often reach over 100%, this implies that both self-healing and continued hydration of the bulk material contribute to the overall stiffness of the self-healing specimens.

The large standard deviations seen in 6a can be attributed to the various levels of healing between specimens. The stiffness of a self-healed specimen is highly dependent on the crack width distribution. Larger cracks will not heal as well and could downgrade the overall stiffness of the specimen. Since all specimens have different crack width distributions after preloading, the level of healing will vary and the stiffness recoveries can vary greatly depending on the size and number of cracks.

Similar to the stiffness recovery results, the first cracking strength recovery also increased as the duration of natural environment exposure increased (Figure 6b). The average recovery levels were near 80% after the first 3 months of natural environment exposure, but then increased to an average of 116% for both the 0.5% and 1.0% preloaded specimens after 12 months of exposure. This high level of recovery is not always seen under laboratory conditions, where first cracking strengths after self-healing are often lower than the first cracking strengths seen during preloading [19,20]. These high levels of recovery are most likely due to the relatively long duration of natural environment exposure, where specimens are exposed to large amounts of precipitation. This causes continued hydration and thus an increase in fracture toughness of the material, which increases the first cracking strength. It should be noted that while recoveries can be over 100%, the value of the first cracking strength of a preloaded specimen upon reloading is not larger than the first cracking strength of a control specimen (no preloading) that was loaded subsequent to the same duration of natural environment exposure.

#### 3.2.2. Effect of Multiple Damage (Reloading) Events on Mechanical Properties

**Figure 7:**
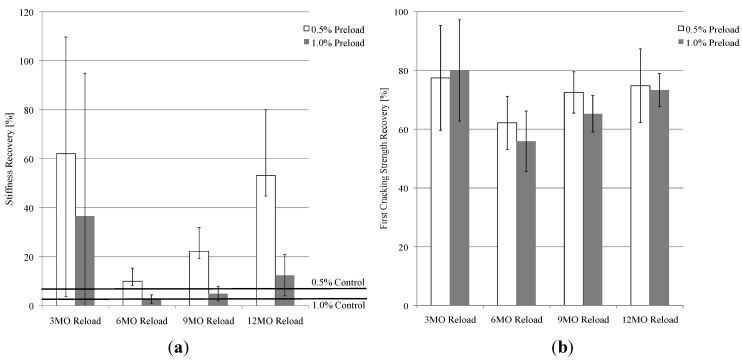
Effect of multiple damage (reloading) events on recovery of mechanical properties. Recovery of (**a**) stiffness and (**b**) first cracking strength. Each data bar is an average of 5 specimens.

As seen in Figure 7, there was no consistently increasing or decreasing trend in the level of stiffness or first cracking strength recovery for specimens (Set 1) that underwent multiple reloading events (at 3, 6, 9, and 12 months). The recovered mechanical properties of the specimens measured by the 6 and 9 month reloading events were lower than those measured by the 3 and 12 month reloading events.

**Figure 8:**
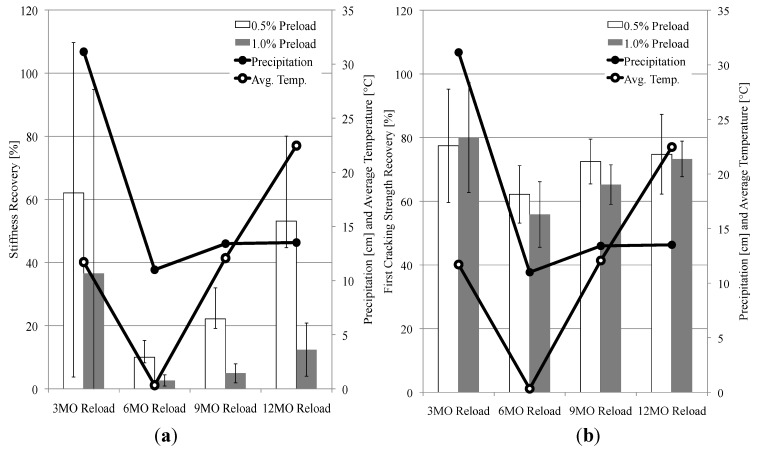
Effect of precipitation and average temperature on self-healing. Recovery of (**a**) stiffness and (**b**) first cracking strength.

It is hypothesized that the extent of recovery may be related to the natural environment conditions experienced by the specimens prior to the reloading events. To test this hypothesis, the total amount of precipitation and the average temperature over each 3-month interval prior to the reloading events were calculated and are shown in Figure 8 (The temperature and precipitation data for Ann Arbor, MI from September 2011 through September 2012 were collected from Weather Underground [33]). There is a reasonably consistent trend between the level of stiffness and first cracking strength recovery and the amount of precipitation, although there is not a significant increase in precipitation to account for the increase in recoveries for the 12-month reloading event. However, there is an extremely consistent trend between the amount of stiffness and first cracking strength recovery and the average temperature. So, while there was no significant increase in the amount of precipitation to account for the increase in stiffness and first cracking strength recovery for the 12-month reloading event, there was a large increase in the average temperature. This suggests that both temperature and the amount of precipitation play an important role in self-healing in the natural environment.

Even with the varying amounts of precipitation and changes in average temperature, both the 0.5% and 1.0% preloaded specimens had average stiffness recovery values larger than the corresponding controls (without healing) shown in Figure 7a for every reloading event. However, the recovery was not 100%. The highest recovery was during the 3-month reloading, 62% for the 0.5% preload and 37% for the 1.0% preload, and the lowest recovery was seen during the 6-month reloading, 10% for the 0.5% preload and 3% for the 1% preloaded specimens. 

Although the recovery of the first cracking strengths did not reach 100%, the level of recovery was still relatively high for every reloading event (Figure 7b). Similar to stiffness recovery, the highest first cracking strength recovery was during the 3-month reloading event (77% for the 0.5% preloaded specimens and 80% for the 1.0% preload) and the lowest recovery was during the 6-month reloading event (62% for the 0.5% preload and 56% for the 1.0% preload).

Since there was recovery of both the stiffness and first cracking strength after each loading cycle, this suggests that self-healing in ECC is repeatable in a multiple imposed damage scenario, but more research is needed to ensure 100% recovery after several loading events.

## 4. Conclusions

Based on the experimental results in this study, the following conclusions can be drawn:

(1) Self-healing of microcracks in ECC can occur in a natural environment despite a high level of damage caused by preloading tensile deformations of 0.5% and 1.0% and wide swings in temperature and precipitationin Michigan climate. Self-healing is not limited to a controlled laboratory environment.

(2) All ECC specimens recovered 95%–105% of the original RF values when exposed to a natural environment for various time periods (up to one year in this study) subsequent to a damage (preloading) event; the observed fluctuations in RF readings during the recording periods may be due to the washout and reformation of healing products caused by large rainfall events.

(3) The level of stiffness and first cracking strength recovery increased with the time of natural environment exposure after the damage (preloading) event, often exceeding 100% recovery values after 6 months of exposure.

(4) For the specimens that underwent multiple loading cycles, the amount of RF recovery decreased after each loading event. However, even after the third damage cycle, the specimens strained to 0.5% were still able to recover to 88% and the specimens strained to 1.0% recovered to 84% of the RF value of control specimens that underwent the same environmental exposure.

(5) In addition to RF recovery, there was recovery in stiffness and first cracking strength after exposure to a natural environment subsequent to each loading event for the specimens that underwent multiple loading cycles. However, the extent of self-healing significantly depends on the temperature and amount of precipitation during the exposure period.

(6) Self-healing functionality can be maintained under multiple damage events. Resonant frequency, stiffness recovery, and first cracking strength recovery data for the samples that underwent multiple loading cycles suggest that self-healing in ECC is repeatable, although deterioration in the recovery magnitude may be expected with each damage load cycle.
